# Anal human papillomavirus and its associations with abnormal anal cytology among men who have sex with men

**DOI:** 10.1038/s41598-020-59967-4

**Published:** 2020-02-21

**Authors:** Ping-Feng Wu, Jen-Fan Hang, Carol Strong, Su-Jung Chen, Li-Ya Lin, Shu-Sheng Chen, Chiung-Ru Lai, Stephane Wen-Wei Ku, Mei-Hsuan Lee

**Affiliations:** 1 Division of Infectious Diseases, Department of Medicine, Taipei Veterans General Hospital, Taipei, 112 Taiwan; 2 School of Medicine, National Yang-Ming University, Taipei, 112 Taiwan; 3 Institute of Clinical Medicine, National Yang-Ming University, Taipei, 112 Taiwan; 4 Department of Pathology and Laboratory Medicine, Taipei Veterans General Hospital, Taipei, 112 Taiwan; 5 Department of Public Health, National Cheng Kung University Hospital, College of Medicine, National Cheng Kung University, Tainan, 701 Taiwan; 6 Division of Infectious Diseases, Department of Medicine, Taipei City Hospital Renai Branch, Taipei, 106 Taiwan

**Keywords:** Infectious diseases, Viral infection

## Abstract

Human papillomavirus (HPV) infection contributes to most anal cancers and premalignant intraepithelial lesions. This study investigated anal HPV infections and cytological abnormalities among men who have sex with men (MSM). Sociodemographic characteristics and sexual behaviors were collected by using a structured questionnaire. Anal cytological results were examined, and HPV genotyping was performed by the Linear Array HPV Genotyping test. Logistic regression was used to estimate risk factors and their associations with high-risk HPV infection and cytological abnormalities. Among 163 MSM, 101 were seropositive for human immunodeficiency virus (HIV) and 62 were seronegative for HIV. The overall prevalence of HPV was 66.2%. A total of 61.9% and 48.2% of participants had never acquired any of either the quadrivalent or nonavalent vaccine HPV types, respectively. Cytological findings showed 15.3% atypical squamous cells of undetermined significance, 16.6% low-grade squamous intraepithelial lesion, 4.9% atypical squamous cells that cannot exclude high-grade squamous intraepithelial lesion and 17% high-grade squamous intraepithelial lesion. The number of high-risk HPV types was the predominant risk factor for abnormal anal cytology (OR 2.02, 95% CI 1.27–3.24). Infection with high-risk HPV was a significant predictor for cytological abnormality. MSM should be encouraged to obtain the HPV vaccine.

## Introduction

Human papillomavirus (HPV) is an important cause of sexually transmitted diseases worldwide and is associated with malignant transformations in the squamous epithelium of the anus^1^. A rising trend in the incidence of anal cancer has been shown in recent years, increasing from 0.8 per 100,000 person-years in 1975 to 1.8 per 100,000 person-years in 2014 in the United States^2^. The development of anal cancer or pre-cancerous lesions is associated with one’s lifetime number of sexual partners, human immunodeficiency virus (HIV) infection, receptive anal intercourse and anal HPV infection, particularly infection with HPV16^3–5^. Moreover, among individuals with anal cancer, nearly 90% of cases are attributable to HPV infection^6^. A recent meta-analysis also showed that the increased prevalence of HPV is associated with the severity of abnormal anal cytology^7^.

The incidence of anal cancer among patients with HIV infection is much higher than among the general population (51.4 *versus* 1.2 per 100,000 person-years)^8^. HIV infection may attenuate the host’s immune competence, which then worsens the control of anal HPV infections^9–11^. In addition, HIV seropositives have a decreased probability of clearing anal HPV infection and have prolonged anal HPV infections, compared to HIV seronegatives^11^. The persistent anal HPV infections have been particularly found in high-risk HPV types, which are the predominant risk factors for anal cancer^12,13^. Thus, HIV infection is considered one of the most important independent risk factors for high-risk anal HPV infection^14–17^.

Anal HPV infection is mainly transmitted through anal intercourse, which is usually considered as a major high-risk sexual behavior in men who have sex with men (MSM)^18^. A previous meta-analysis found that the prevalence of anal HPV infection and epithelial dysplasia among MSM greatly exceeded the prevalence in the cervix among women^3^. Moreover, among the HIV infected population, the prevalence of any anal HPV type was 96% among MSM, 90% among women and 59% among men who have sex with women, suggesting that the acquisition of anal HPV infection is dependent on sexual behaviors^19^. However, most of the previous studies were mainly confined to HIV seropositive MSM. Data comparing HPV prevalence among both HIV seropositive and seronegative MSM is limited.

Therefore, this study aimed to evaluate the associations of HPV infection with the risk of abnormal anal dysplasia among MSM. Moreover, the different types or numbers of HPV infections were estimated by stratifying by HIV seronegatives and seropositives.

## Results

There were 163 MSM enrolled in this study; 101 were HIV seropositive, and 62 were HIV seronegative. Table 1 shows the baseline characteristics of the study participants. The median age among HIV seropositives was 32 years (interquartile range, IQR, 28–38 years old) compared to 29 years (IQR, 26–34 years old) among HIV seronegatives (*p* = 0.004). HIV seropositives had a higher percentage of smokers than HIV seronegatives (25.7% *versus* 8.1%, *p* = 0.010). Among HIV seropositives, 78.2% had undetectable HIV RNA. In addition, the median CD4^+^ cell count was 551 cells/µl (IQR, 430–730 cells/µl), and the median CD4^+^ nadir cell count was 251 cells/µl (IQR, 160–379 cells/µl).

**Table 1:** Baseline demographic and clinical characteristics of 163 participants, stratified by HIV status.

Baseline characteristics	Total, n = 163	HIV-positive MSM n = 101	HIV-negative MSM n = 62	*p* value
		No. (%)	No. (%)	
Age (years), median (IQR)	31.0 (27.0–36.0)	32.0 (28.0–38.0)	29.0 (26.0–34.0)	0.004
Cigarette smoking	31 (19.0)	26 (25.7)	5 (8.1)	0.010
**Partnership status**				
Single/fixed partner	150 (92.0)	92 (91.1)	58 (93.5)	0.768
Educational status				
College or above	151 (92.6)	93 (92.1)	58 (93.5)	1.000
**Income per month**				
≥1300 USD	74 (45.4)	47 (46.5)	27 (43.5)	0.834
Previous HPV vaccination	19 (11.7)	10 (9.9)	9 (14.5)	0.522
Circumcision	33 (20.2)	19 (18.8)	14 (22.6)	0.704
No insertive anal intercourse within 1 year	24 (14.7)	12 (11.9)	12 (19.4)	0.280
No receptive anal intercourse within 1 year	23 (14.1)	10 (9.9)	13 (21.0)	0.082
**Inconsistent condom use**				
During insertive anal intercourse	75 (54.0)	45 (50.6)	30 (60.0)	0.371
During receptive anal intercourse	79 (56.4)	47 (51.6)	32 (65.3)	0.169
**>5 sexual partners within 1 year**				
Insertive anal intercourse	63 (38.7)	36 (35.6)	27 (43.5)	0.401
Receptive anal intercourse	50 (30.7)	28 (27.7)	22 (35.5)	0.385
STI within 1 year	72 (44.2)	51 (50.5)	21 (33.9)	0.056
**HIV-related factors**				
Viral load <40 copies/ml		79 (78.2)		
CD4^+^ cell count(cells/µl), median (IQR)		551 (430–730)		
Nadir CD4^+^ cell count (cells/µl), median (IQR)		251 (160–379)		

Human immunodeficiency virus (HIV). Human papillomavirus (HPV). Interquartile range (IQR). Men who have sex with men (MSM). Sexually transmitted disease (STD). United States dollar (USD).

**Figure 1:**
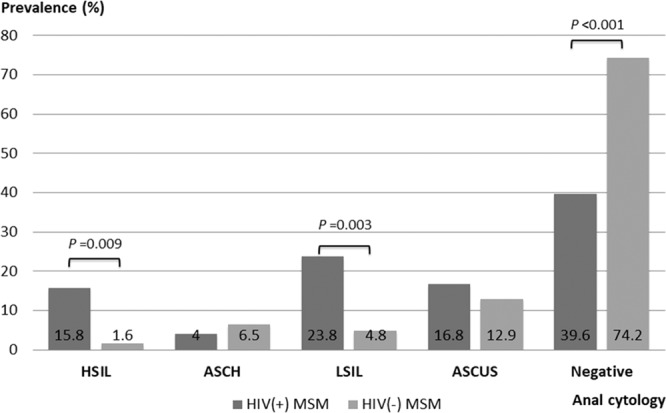
Prevalence of anal cytology results among men who have sex with men with and without human immunodeficiency virus infection.

All study participants received anal cytology examinations. Figure 1 shows the anal cytology findings. Among our study participants, there were 86 (52.8%) normal cytological findings, 25 (15.3%) atypical squamous cells of undetermined significance (ASCUS), 27 (16.6%) low-grade squamous intraepithelial lesion (LSIL), 8 (4.9%) had atypical squamous cells that cannot exclude high-grade squamous intraepithelial lesion (ASCH) and 17 (10.4%) high-grade squamous intraepithelial lesion (HSIL). When compared to HIV seronegatives, HIV seropositives had a higher frequency of abnormal anal cytology results (*p* < 0.001). Compared to HIV seronegatives, the prevalence of LSIL (23.8% *versus* 4.8%, *p* = 0.003) and HSIL (15.8% *versus* 1.6%, *p* = 0.009) were significantly higher among HIV seropositives.

Table 2 shows the baseline characteristics among participants with normal or abnormal anal cytology. Participants with abnormal anal cytology tended to be older, seropositive for HIV and had more receptive anal intercourse within one year (*p* < 0.05). In addition, participants with abnormal anal cytology showed a prevalence of 23.5% and 17.6% for HPV16 and HPV18 infection, respectively, compared to 5.6% and 2.8% for HPV16 and HPV18 infections among those with normal anal cytology (*p* < 0.01).

**Table 2:** Anal intraepithelial neoplasia among 163 participants.

Characteristics	Normal cytology n = 86	Abnormal cytology n = 77	*p* value
	No. (%)	No. (%)	
Age, median (IQR), years	29.5 (26.0–35.0)	33.0 (28.0–37.0)	0.015
Positive HIV status	40 (46.5)	61 (79.2)	<0.001
Cigarette smoking	14 (16.3)	17 (22.1)	0.458
**Partnership status**			
Single/Fixed partner	80 (93.0)	70 (90.9)	0.835
**Education status**			
College or above	82 (95.3)	69 (89.6)	0.271
**Income**			
≥1300 USD/month	36 (41.9)	38 (49.4)	0.423
Previous HPV vaccination	11 (12.8)	8 (10.4)	0.816
Circumcision	18 (20.9)	15 (19.5)	0.972
No insertive anal intercourse within 1 year	12 (14.0)	12 (15.6)	0.943
No receptive anal intercourse within 1 year	17 (19.8)	6 (7.8)	0.049
**Inconsistent condom use**			
During insertive anal intercourse	43 (58.1)	32 (49.2)	0.380
During receptive anal intercourse	38 (55.1)	41 (57.7)	0.882
**>5 sexual partners within 1 year**			
Insertive anal intercourse	33 (38.4)	30 (39.0)	1.000
Receptive anal intercourse	25 (29.1)	25 (32.5)	0.765
STI within 1 year	34 (39.5)	38 (49.4)	0.271
**HPV DNA detection**			
Number of detected high-risk HPV genotypes	0 (0–1)	1 (0–3)	<0.001
Detection of HPV16	4/71 (5.6)	16/68 (23.5)	0.006
Detection of HPV18	2/71 (2.8)	12/68 (17.6)	0.009

Human immunodeficiency virus (HIV). Human papillomavirus (HPV). Interquartile range (IQR). Sexually transmitted disease (STD). United States dollar (USD).

As shown in Table 3, HIV seropositivity and number of detected high-risk HPV showed significant positive associations with anal intraepithelial neoplasia (*p* < 0.001). On the other hand, the absence of receptive anal intercourse within one year showed negative associations with abnormal anal cytology (*p* = 0.034). In the multivariate model, only the number of detected high-risk HPV types was significantly associated with abnormal anal cytology, with an adjusted OR of 2.02 (1.27–3.24).

**Table 3:** Factors associated with anal intraepithelial neoplasia among 163 participants.

Characteristics	Univariate Analysis		Multivariate Analysis	
	Odds ratio (95% CI)	*p* value	Odds ratio (95% CI)	*p* value
Age	1.06 (1.01–1.11)	0.022	1.05 (0.99–1.13)	0.112
Positive HIV status	4.38 (2.19–8.78)	<0.001	2.04 (0.83–4.98)	0.120
No receptive anal intercourse within 1 year	0.34 (0.13–0.92)	0.034	0.32 (0.08–1.20)	0.090
**HPV DNA detection**				
Number of detected high-risk HPV genotypes	2.50 (1.68–3.71)	<0.001	2.02 (1.27–3.24)	0.003
Detection of HPV16	5.15 (1.63–16.34)	0.005	1.18 (0.25–5.55)	0.831
Detection of HPV18	7.39 (1.59–34.41)	0.011	1.39 (0.22–8.70)	0.725

Human immunodeficiency virus (HIV). Human papillomavirus (HPV).

**Figure 2:**
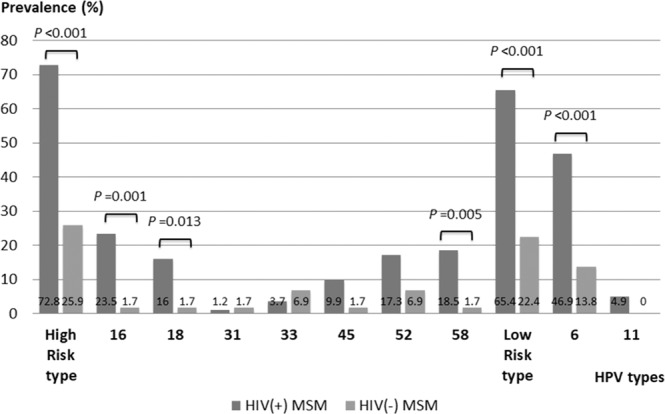
Prevalence of various types of anal human papillomavirus infection among men who have sex with men, with and without human immunodeficiency virus infection.

There were 139 (85.3%) participants that had sufficient anal samples for HPV genotyping. Among these men, 81 (58.3%) were HIV seropositive whereas 58 (41.7%) were HIV seronegative. Figure 2 displays the distribution of infection with different HPV types. Among the 37 types of HPV, 66.2% (92/139) of the study participants tested positive for at least one, including 20.9% (29/139) for one HPV type, 13.7% (19/139) for two HPV types, 12.2% (17/139) for three HPV types, 7.2% (10/139) for four HPV types and 12.2% (17/139) for more than four HPV types. Forty-five percent (63/139) had acquired more than one type of anal HPV infection. Multiple HPV infections were more prevalent among HIV seropositives compared to HIV seronegatives (69.1% *versus* 12.1%, *p* < 0.001). The overall prevalence of high-risk and low-risk HPV infection was 53.2% (74/139) and 47.5% (66/139), respectively. Compared to HIV seronegatives, HIV seropositives had a significantly higher prevalence of both of high- and low-risk HPV infection types (72.8% *versus* 25.9%, *p* < 0.001 for high-risk types and 65.4% *versus* 22.4%, *p* < 0.001 for low-risk types). HPV16 (27.0%) and HPV6 (69.7%) were the most common high-risk and low-risk HPV type, respectively. Both were more frequently observed among HIV seropositives than among seronegatives (23.5% *versus* 1.7%, *p* < 0.001 for HPV16 and 46.9% *versus* 13.8%, *p* < 0.001 for HPV6). We also examined the risk factors associated with high-risk HPV infections (Table 4). In multivariate analyses, HIV seropositivity, having more than five receptive intercourse partners within one year and a history of sexually transmitted diseases within one year were positively associated with high-risk HPV infections (*p* < 0.05). Similarly, HIV seropositivity was an important associated factor for HPV16 and HPV18 infections with the adjusted OR of 21.3 (2.37–192.40) (*p* = 0.006) and 13.60 (1.54–120.36) (*p* = 0.019), respectively. There were 13.7% (19/139) participants without any receptive sexual partner, 56.8% (79/139) participants with one to five receptive sexual partners and 29.5% (41/139) participants with above five receptive sexual partners. There were 43.9% (61/139) participants with a history of sexually transmitted diseases (38.1% with one, 3.6% with two, 0.7% with three, 0.7% with four and 0.7% with five). On the other hand, HIV seropositivity was the only risk factor associated with low-risk HPV infections (OR 7.00, 95% CI 3.07–15.95).

**Table 4:** Factors associated with high- or low-risk anal HPV types among 139 participants.

Characteristics	Univariate analysis		Multivariate analysis	
	Odds ratio (95% CI)	*p* value	Odds ratio (95% CI)	*p* value
**High-risk anal HPV infection**				
Age, median (IQR), years	1.05 (1.00–1.10)	0.072	1.04 (0.97–1.11)	0.292
Positive HIV status	7.69 (3.58–16.52)	<0.001	6.60 (2.59–16.82)	<0.001
Cigarette smoking	3.40 (1.26–9.13)	0.015	2.09 (0.62–6.97)	0.233
Circumcision	0.34 (0.14–0.86)	0.022	0.39 (0.12–1.23)	0.108
>5 receptive anal sexual partners within 1 year	2.09 (0.98–4.46)	0.056	3.37 (1.20–9.45)	0.021
STI within 1 year	6.34 (2.97–13.53)	<0.001	5.18 (2.15–12.45)	<0.001
**Low-risk anal HPV infection**				
Age, median (IQR), years	1.01 (0.96–1.06)	0.754	0.97 (0.92–1.03)	0.264
Positive HIV status	6.55 (3.04–14.13)	<0.001	7.00 (3.07–15.95)	<0.001
Cigarette smoking	2.28 (0.93–5.58)	0.072	1.44 (0.54–3.84)	0.468

Human immunodeficiency virus (HIV). Human papillomavirus (HPV). Interquartile range (IQR). Men who have sex with men (MSM). Sexually transmitted disease (STD).

The prevalence of vaccine-preventable HPV types among the 139 MSM is shown in Table 5. There were 86 (61.9%) and 67 (48.2%) participants that had never acquired any of the HPV types included in the quadrivalent or nonavalent HPV vaccine. Among our study participants, none had acquired all four quadrivalent vaccine HPV types or more than four nonavalent vaccine HPV types. There were significantly fewer detected quadrivalent vaccine types in HIV seronegatives than in seropositives (15.5% *versus* 54.3%, *p* < 0.001). The same pattern was seen for nonavalent vaccine HPV types (25.9% *versus* 70.4%, *p* < 0.001).

**Table 5:** Prevalence of vaccine-preventable HPV in 139 MSM.

	Total n (%)	HIV-negative n (%)	HIV-positive n (%)	*p* value
**Number of quadrivalent vaccine HPV types (HPV6/11/16/18)**				
0	86 (61.9)	49 (84.5)	37 (45.7)	<0.001
1	27 (19.4)	8 (13.8)	19 (23.5)	0.229
2	21 (15.1)	1 (1.7)	20 (24.7)	<0.001
3	5 (3.6)	0 (0.0)	5 (6.2)	0.075
4	0 (0.0)	0 (0.0)	0 (0.0)	—
**Number of nanovalent vaccine HPV types (HPV6/11/16/18/31/33/45/52/58)**				
0	67 (48.2)	43 (74.1)	24 (29.6)	<0.001
1	32 (23.0)	10 (17.2)	22 (27.2)	0.244
2	23 (16.5)	4 (6.9)	19 (23.5)	0.018
3	10 (7.2)	1 (1.7)	9 (11.1)	0.045
4	7 (5.0)	0 (0.0)	7 (8.6)	0.041
5	0 (0.0)	0 (0.0)	0 (0.0)	—
6	0 (0.0)	0 (0.0)	0 (0.0)	—
7	0 (0.0)	0 (0.0)	0 (0.0)	—
8	0 (0.0)	0 (0.0)	0 (0.0)	—
9	0 (0.0)	0 (0.0)	0 (0.0)	—

Human immunodeficiency virus (HIV). Human papillomavirus (HPV). Men who have sex with men (MSM).

## Discussion

Anal HPV infection is primarily acquired through sexual exposure, especially via anal intercourse. HPV infection contributes substantially to the risk for anal cancer and squamous intraepithelial lesions. However, there is still limited epidemiological studies investigating the association between anal HPV infection and anal intraepithelial lesions among MSM regardless of their HIV infection status, particularly among the Asian population. The study was conducted in a large medical center which provides substantial healthcare for HIV-seropositives as well as pre- or post-exposure prophylaxis for HIV-seronegatives. Because MSM are considered as a high risk group for HIV infection^20,21^, the participants enrolled in this study should be representative of most of the MSM in northern Taiwan with considerable generalizability. This study showed that both high- and low-risk anal HPV infections were significantly more prevalent in HIV seropositives than in HIV seronegatives. Individuals with increased numbers of acquired HPV types had increased odds of abnormal anal cytology. However, not many individuals (15.5% of HIV seronegatives and 54.3% of HIV seropositives) were infected with at one or more of the quadrivalent vaccine types (HPV6, 11, 16, 18), suggesting that the HPV vaccine should be advised in these individuals.

We found that the prevalence of anal HPV infection was 85.2% in HIV seropositives and 39.7% in HIV seronegatives. This HPV prevalence is comparable with other studies conducted in Asian countries^17,22,23^. Among MSM seropositive for HIV, the prevalence of HPV infection was 82.7% in China, 85% in Thailand and 85.3% in Taiwan^17,22,23^. On the other hand, the prevalence was even higher in Western countries, showing 97% in the United States and 96.3% in Italy^24,25^. Compared to MSM that were seropositive for HIV, those who were HIV seronegative had consistently lower prevalence of anal HPV infection, which was reported to be 58.5–73.3% and 70% in Asian and Western countries, respectively^17,22–25^. Another recent study among MSM of various ethnicities suggested that the estimated global prevalence of anal HPV infection among HIV seropositives and seronegatives was 79% and 47%, respectively^26^.

In terms of HPV types, the most common types of high-risk genital HPV among HIV seropositive MSM in Taiwan were HPV16 (5.9%), HPV51 (7.9%) and HPV52 (7.2%)^20^. In Western countries, the most common types was HPV16, with a prevalence of 29.0%^27^. However, the prevalence of HPV52 was around 10%^7,27^. Our study found the prevalence of HPV16 and HPV52 to be 23.5% and 17.3%, suggesting that anal HPV52 infection among MSM is more prominent and increasing in Taiwan. A majority of anal cancer was attributed to HPV infections^6^; nevertheless, the impact of different high-risk HPV types in precancerous lesions on anal cancer has not been clearly identified. The importance of anal HPV52 infection in Taiwan might be worth further investigation.

MSM and increased numbers of acquired high-risk HPV types were found to significantly increase the risk for anal atypical squamous lesions in recent reports^19,21,28,29^. On the other hand, a meta-analysis suggested that HPV16 infection, but not the number of acquired high-risk HPV types was associated with increased risk for anal cancer^7^. This may be due to the fact that the study included different populations, such as HIV seropositive male and female patients regardless of their sexual orientation^7^.

In our study, we found that the prevalence of high-risk HPV infection, including HPV16, HPV31, HPV51, HPV56, HPV58 and HPV68, increased with the severity of abnormal anal cytological findings. The prevalence of high-risk HPV was 35.2% for normal anal cytology, 68.9% for LSIL and ASCUS and 78.3% for ASCH and HISL. These findings are consistent with previous reports^7,27^. Interestingly, among those with high-risk HPV infection, the numbers of detected high-risk HPV types also increased with the severity of abnormal anal cytology. The median numbers of detected high-risk HPV infections was 0 (IQR, 0–1) for individuals with normal anal cytology, 1 (IQR, 0–2.5) for ASCUS, 1.5 (IQR, 1–3) for LSIL, and 2 (IQR, 1–3) for ASCH and HSIL. Notably, HIV serostatus was also significantly positively associated with HPV infection. HIV seropositives who are immunosuppressed may be more prone to persistent HPV infection, therefore increasing their risk for anal cancer^17,20^. However, given the multitude of HPV types, the impact of multiple HPV infections on anal dysplasia needs further investigation in large-scale studies.

Most anal cancers or precancerous HSIL among MSM are attributed to HPV infection, especially infection via anal intercourse^6,18,28,30,31^. Strategies for primary prevention of HPV infection are crucial for the control of anal dysplasia or anal cancer among MSM. Our study found fewer detected quadrivalent or nonavalent vaccine HPV types among HIV seronegatives than among HIV seropositives. Moreover, 61.9% of our study population did not yet acquire any of the HPV types covered in quadrivalent vaccines (HPV6/11/16/18). Thus, this study suggests that there is still a substantial proportion of MSM that may benefit from HPV vaccination. Therefore, increasing awareness of HPV vaccines may be a practical strategy for prevention among MSM, as HPV vaccines have also been suggested for MSM in previous studies^21,32^.

There are limitations to this study that should be considered. Due to its cross-sectional design, it may be difficult to infer causality, since we could not establish causal temporality. Although we found that HIV seropositivity was associated with acquisition of both low-risk and high-risk HPV types, it was not clear whether HIV infection preceded the HPV infection or not. It is possible that individuals with HIV infection were unable to clear HPV because of impaired immunity. On the other hand, there is also the possibility that individuals with HPV infection may be more sexually active, therefore making them more vulnerable to HIV infections. Lastly, due to the heterogeneity in HPV types, larger sample sizes are needed to further elucidate the influence of specific HPV types or number of anal HPV infections on abnormal anal cytological lesions.

In conclusion, we found a high prevalence of anal dysplasia and HPV infection among MSM regardless of their HIV serostatus. Individuals with increased numbers of HPV infections had stronger associations with anal dysplasia. In addition, there were still a substantial number of MSM who had not yet acquired any of the HPV types that are preventable by vaccines. Therefore, MSM should be advised to receive HPV vaccinations.

## Methods

### Study design and subjects

This cross-sectional study was conducted in a tertiary medical center, Taipei Veterans General Hospital in northern Taiwan. Males at least 20 years of age who reported having regular sex with men in the past 6 months were enrolled from March, 2015 to July, 2016. The MSM who were HIV seropositive were recruited from outpatient clinics during their regular follow-ups. On the other hand, the MSM who were seronegative for HIV were recruited from voluntary HIV testing clinics. These men were confirmed as HIV-negative by Western blot using the recomline HIV-1 & HIV-2 IgG (Mikrogen GmbH, Neuried, Germany) or HIV viral load examination using cobas HIV-1 (Roche Diagnostics, Mannheim, Germany). Participants with a history of anal cancer or abnormal anal cytology were excluded. All methods in this study were carried out in accordance with the principles of the Declaration of Helsinki. This study was approved by the Institution Review Board of Taipei Veterans General Hospital. All of the study subjects provided informed consent.

### Data collection

Study subjects were interviewed at study entry using a structured questionnaire (Supplementary questionnaire). The information collected included demographic characteristics (age, educational levels and income), smoking status, partnership (single or fixed partner), sexual behavior within 1 year, HPV vaccination status and history of sexually transmitted diseases. Clinical data on HIV infection at study entry was obtained by medical chart review with a standardized form. Data on HIV viral load, CD4^+^ cell count and nadir CD4^+^ cell count was abstracted.

### Anal specimen collection for cytology and HPV genotyping

At the enrollment visit, well-trained health-care professionals sampled anal specimens from all participants using LIBO Specimen Collection and Transport Swab (LIBO Medical Products Inc., New Taipei City, Taiwan) with 5 cm insertion into the anal canal and a slow 360-degree rotating extraction. The swab was then shaken in SurePath liquid-based cytology fluid (Becton, Dickinson and Company, NJ, USA). The anal cytology slides were screened and labeled by cytotechnologists first then interpreted by two cytopathologists separately blinded for HIV or HPV infection status. Slides with discordant interpretations were reviewed again under a multi-head microscope for agreements. The cytological results were interpreted according to the Bethesda System terminology as negative, ASCUS, LSIL, ASCH and HSIL^33^. The remaining samples were sent for HPV genotyping using the Linear Array HPV Genotyping test (Roche Molecular Systems, Inc., Pleasanton, CA), per the manufacturer’s instructions. Briefly, the target DNA was first amplified using polymerase chain reaction. Then, the nucleic acid was hybridized to identify 37 anal HPV DNA genotypes, including high-risk types (16, 18, 26, 31, 33, 35, 39, 45, 51, 52, 53, 56, 58, 59, 66, 68 and 82) and low-risk types (6, 11, 40, 42, 54, 61, 70, 72, 81 and CP6108)^34^. The Human ß-globin gene was used as a primer for internal control.

### Statistical analysis

We compared baseline characteristics between HIV seropositive and seronegative MSM and tested differences in categorical variables using Chi-squared or Fisher’s exact tests, while continuous variables using Student-t or Mann-Whitney *U* tests. We then used logistic regression models to estimate the associations of baseline characteristics (age, HIV seropositivity, receptive anal intercourse, detectable HPV) with intraepithelial neoplasia. In addition, we also investigated possible risk factors associated with high-risk or low-risk HPV types. Odds ratios (ORs) with 95% confidence intervals (CIs) were obtained from the logistic regressions. Two-tailed tests were used to determine statistical significance and a value of *p* < 0.05 was considered significant. All statistical analyses were performed using SPSS software version 20 (SPSS INC, Chicago, IL, USA).

## Supplementary information

Supplementary information.
