# Supplementary material for: Anal human papillomavirus and its associations with abnormal anal cytology among men who have sex with men
Source: Sci Rep. 2020 Feb 21;10:3165. doi: 10.1038/s41598-020-59967-4 (PMC7035349; doi:10.1038/s41598-020-59967-4)
Supplement: Supplementary file 1 — Supplementary information. [file 41598_2020_59967_MOESM1_ESM.pdf]

Part 1、Basic Information

1. Your birthday

   ____

2. Object of sexual behavior？
   【 】Male
   【 】Female
   【 】Both female and male

3. Current relationship status:
   【 】Single
   【 】Fixed partner
   【 】Open relationship

4. Educational levels:
   【 】Elementary school
   【 】Junior high school
   【 】Senior high school or vocational high school
   【 】University or College
   【 】Graduate institute or above

5. Average income per month in the past year
   【 】No income
   【 】Less than 650 US dollars
   【 】650 US dollars ~ Less than 1300 US dollars
   【 】1300 US dollars ~ Less than 1950 US dollars
   【 】1950 NT dollars ~ Less than 3250 US dollars
   【 】More than 3250 US dollars
   (NT dollars was converted to US dollars according to the exchange rate in May, 2015)

6. Have you ever been vaccinated against HPV previously?
   【 】Yes
   【 】No

Part 2、Sexual behaviors

7. In the past one year, how many sexual partners with whom you had insertive anal intercourses?
   【 】0
   【 】1–5
   【 】More than 5

8. In the past one year, do you have inconsistent condom use during insertive anal intercourses?
   【 】Yes
   【 】No

9. In the past one year, how many sexual partners with whom you had receptive anal intercourses?
【 】0
【 】1–5
【 】More than 5

10. In the past one year, do you have inconsistent condom use during receptive anal intercourses?
【 】Yes
【 】No

Part 3、Sexually transmitted diseases and others

11. In the past one year, have you ever been diagnosed with sexually transmitted diseases
【 】Yes
【 】No

12. What kind of sexually transmitted diseases?
【 】Never being diagnosed with sexually transmitted diseases
【 】Hepatitis C
【 】Neisseria gonorrhea or Chlamydia trachomatis
【 】Syphilis
【 】Condyloma acuminatum
【 】Genital herpes
【 】Other：________________

13. Do you have circumcision?
【 】Yes
【 】No

14. Do you have cigarette smoking?
【 】Yes
【 】No
